# Barriers and facilitators to access sexual and reproductive health services among young migrants in Tarapacá, Chile: a qualitative study

**DOI:** 10.1186/s12889-024-17884-5

**Published:** 2024-02-05

**Authors:** Alexandra Obach, Alice Blukacz, Michelle Sadler, Alejandra Carreño Calderón, Báltica Cabieses, Carolina Díaz

**Affiliations:** 1 Centro de Salud Global Intercultural, Instituto de Ciencias e Innovación en Medicina, Facultad de Medicina Clínica Alemana, y Facultad de Psicología, Universidad del Desarrollo, Santiago, Chile; 2 Departamento de Historia y Ciencias Sociales, Facultad de Artes Liberales, Universidad Adolfo Ibáñez, Santiago, Chile

**Keywords:** Reproductive health, Sexual health, Access, Healthcare services, Migration, Youth, Chile

## Abstract

**Background:**

Chile has become a destination country for immigrants from Latin America, including youth. Guaranteeing access and use of sexual and reproductive health services for young migrants is crucial because of their overlapping experiences of transitioning to a new country and to adulthood. However, the existing evidence shows barriers to accessing sexual and reproductive healthcare among young migrant populations. In this context, the main objective of this article is to identify the barriers and facilitators that young migrants experience to access sexual and reproductive healthcare in the Tarapacá region of Chile.

**Methods:**

A qualitative study was conducted in the Tarapacá region of Chile. Semi-structured interviews with 25 young migrants from Venezuela, Colombia, and Ecuador, as well as 10 health workers, were carried out. The interviews were transcribed and thematically analysed. The study was approved by the Ethics Committee of the Universidad del Desarrollo (#2019-22).

**Results:**

Young migrants face barriers linked to structural shortcomings within the healthcare system, which may be similar to those faced by the local population. Barriers are also derived from reductionist sexual and reproductive health approaches, which prioritise the prevention of pregnancy, sexually transmitted infections, and HIV, with a predominantly heteronormative focus. The prevailing narratives from the health system are those of risk and lack of control and self-care among young people, and they are exacerbated in the case of migrants. Young migrants, especially from the Caribbean, are stereotyped as over-sexualised and liberal in comparison to the local population and believed to be engaging in riskier sexual behaviours that should be kept under check. This may translate into experiences of discrimination and mistreatment when receiving care. Facilitators include good-quality information and community-level interventions.

**Conclusions:**

This study shows a limited approach to the sexual and reproductive health of young migrants in Chile, severely hampering their reproductive and sexual rights. Policies and initiatives must work towards removing structural barriers, changing narratives, and empowering young migrants regarding their sexual and reproductive health.

## Background

During the last decades, Chile has increasingly become a destination for international migrants, especially from other Latin American countries. In 2020, people born abroad represented 8% of the country’s population [1], of which around a third were reported to be young people (15-29 years old) [2]. This figure has likely increased since then, given the large number of people entering the country through non-authorised crossing points during the past 3 years -due to more constrictive laws for migrants to enter the country and the mobility restrictions that were placed during the COVID-19 pandemic [3–6]. Most of these crossing points are located in the northern regions -where the Tarapacá region is located. In 2021, 73,030 international migrants were reported to live in the region, representing a 5.3% increase since 2020 despite border closures [7].

Given the growing migrant population in Chile, policies have been progressively implemented to promote and facilitate their access to health care, such as the 2016 decree granting free health care coverage to undocumented migrants and the 2018 Migrant Health Policy [8, 9]. However, in 2020, 11% of international migrants were not registered for health care coverage, almost three times more than locals [10]. Furthermore, international migrants who experience structural vulnerability face greater exposure to social exclusion, marginalisation and limited access to health services, both in transit and upon arrival in the host country [11–14]. Barriers to access healthcare reported by international migrants in Chile include lack of compliance with the decree mentioned earlier by public healthcare centres, lack of culturally and linguistically relevant information for migrant communities on their right to health and on where to seek health care, as well as administrative barriers such as extended waiting times and limited available time for medical appointments [11, 12, 14, 15]. Specifically, young migrants might encounter difficulties finding accurate information on accessing sexual and reproductive health (SRH) services; they may fear experienced or anticipated discrimination; or those who are undocumented might be afraid of accessing the health system due to possible deportations [11, 12, 14].

Additionally, adolescents from Caribbean countries in Chile have reported “othering” discourses, where stereotyped perceptions of culture are used to explain different behaviours or opinions [16, 17]. These perceptions, discourses and experiences are inscribed in persistent dynamics of racism in Chilean society concerning international migrants [16], who are constructed in the social imaginary as a threat. This social construction is part of the racial hierarchisation operating in Chile, giving migrants from Latin American countries -which are seen as “less developed” than Chile- a lower status [17, 18].

Studies in countries such as Colombia and Brazil [19] have shown the impact that migratory trajectories and living conditions have on the SRH needs of young people, highlighting a lack of knowledge about sexual and reproductive rights and a lack of access to family planning methods, STI prevention, voluntary termination of pregnancy, as well as to essential menstrual health goods. They also face exposure to violence and difficulties in the exercise of free, safe, and pleasurable sexuality. These difficulties have been observed in different countries of the continent and have increased during the COVID-19 pandemic, when the increase in migrant populations entering through unauthorised crossing points was added to the interruption of SRH services [20]. Young and adolescent women are a group of particular concern, as they may face exacerbated social vulnerability during migration processes, compounded with the poverty and violence experienced in their country of origin [21, 22]. In the case of the migratory corridors in Central America, sexual violence is frequently suffered by adolescents and young people. The presence of criminal groups in countries of origin and migratory corridors worsens the situations of violence and extortion suffered by girls, adolescents and young people who are considered “exchange goods” This is also one of the drivers of internal and international migration of adolescents and young people. Finally, studies have shown the situation of LGBTIQ+ groups migrating across the continent and present additional needs related to the treatment of STIs, psychosocial care and support for violence, consumption of psychoactive substances, family conflicts, survival sex, and interruption of gender transition processes [19, 23].

Guaranteeing access and use of SRH services for young migrants is crucial because of their overlapping experiences of transitioning to a new country and to adulthood. These experiences may involve reproductive and sexual rights violations impacting their health and requiring care, including unwanted pregnancies and sexual and gender-based violence [24, 25]. However, the existing evidence shows that many young migrants are not using SRH services due to the shame and stigma attached, including being represented as spreading infectious diseases, such as HIV [26]. Regarding reproductive health, migration and pregnancy have been constructed as particularly problematic in receiving countries, for instance, surrounding “anchor babies”, a pejorative term used to define children of irregular migrants born in countries with an *ius solis* rule regarding citizenship [27, 28]. Additionally, the idea that migrants have more children than their local counterparts, leading to a so-called “replacement” threat, has become popular in political discourses [29].

The evidence shows that reproductive health is a central issue for young migrant women. In Chile, the leading cause of hospital discharges in migrants is pregnancy, childbirth, and the postpartum period. In 2020, in the Tarapacá region, these represented 39% of all hospital discharges of migrant children and adolescents versus 5% among the local population. Although in 2021, these figures were considerably reduced, in migrants, they remained double those of the local population (11% versus 6%) [2]. The consequence is that SRH care for migrants in Latin America has been heavily focused on women’s reproductive issues, leaving aside sexual health, with a significant concentration of studies on maternity, maternal and child health and pregnancy control and outcomes [20, 30–32].

As international migrants are framed as “risky” or “at-risk” populations, the predominant approach to their SRH and rights tends to focus on individual, behavioural, and cultural aspects, failing to address the broader structural forces and the barriers that institutions and health systems place on the promotion of their wellbeing [33, 34]. This is especially the case when addressing the SRH of young migrants, where the predominant risk approach when addressing youth overlaps with that of migrants, especially those facing high degrees of vulnerability [35]. Lupton defines risk as a pivotal discourse in normalisation strategies in the face of potential deviations from the norm. Thus, being described as “high risk” implies being singled out as requiring expert advice, surveillance, and self-regulation [36]. This definition is particularly relevant regarding SRH among migrant youth, given that migration, youth, and SRH are three conceptual areas discursively associated with risk and disciplining [36].

In this context, promoting SRH equity requires empowering individuals and communities and guaranteeing reproductive and sexual rights, considering the particularities of migrant youth. There are few studies in Latin America and Chile regarding the SRH of young migrants. In this article, we seek to identify the barriers and facilitators that young migrants experience to access sexual and reproductive healthcare in the Tarapacá region of Chile to contribute to the existing literature on the topic, which is useful for policymakers and other stakeholders looking to develop initiatives on the issue.

## Methods

This methods section follows the consolidated criteria for reporting qualitative studies (COREQ) 32-item checklist [37].

### Theoretical framework and study setting

This article presents data from a sub-study that is part of a larger research project focused on sexuality, corporality, and HIV/AIDS among youth, including young migrants. The larger project consisted of a qualitative study where the researchers sought to analyse the perceptions of young people and healthcare teams regarding SRH in three regions of Chile. The reason for selecting this study design is that it enables an in-depth understanding of the participants’ experiences, their context, and the social processes regarding SRH in the context of youth and human mobility [38].

Within the larger study design, the sub-study reported in this article was carried out in the Tarapacá Region, located in northern Chile, with a focus on young migrants. This region was selected as it shares a border with Bolivia. Although it has historically been an important entry point for Peruvian and Bolivian migrants, in the past few years, it has experienced increased immigration flows from other Latin American countries such as Venezuela, Colombia, Ecuador, and Haiti. After entering Chile, migrants usually carry on to Iquique, the capital city of the Tarapacá region, and either settle there or move South to other cities, including the capital city of Santiago [39]. Those who stay in Tarapacá represent 4.5% of international migrants in Chile, placing it as the fourth region of settlement after Santiago, Antofagasta, and Valparaíso [2]. Of the young population in the region (18-29 years old), 9.1% were migrants in 2022 [40].

### Participant selection and recruitment

A total of 35 people participated in the sub-study: 25 international young migrants and 10 health workers. Data was collected between June 2021 and June 2022 in Iquique, the capital city of the Tarapacá region, and in Alto Hospicio and Pozo Almonte, two small towns of the same region.

The health workers recruited worked in public health services in the Tarapacá region with migrant populations at the time of study. The first three contacts were recruited through key informants, and the following were either identified by snowball sampling of interviewees or were approached directly in public sector primary healthcare centres (Family Healthcare Centres, CESFAM thereafter) settled in districts with high migrant populations. The final sample included five midwives, three social workers, one physician, and one psychologist working in CESFAMs.

The young migrants recruited were living in the Tarapacá Region and had been born in Venezuela, Colombia, and Ecuador. These countries were chosen because large scale migration from their territories to Chile is a recent phenomenon, and, therefore, very little is known about youth’s perceptions of SRH and their access to the Chilean health system. Recruitment occurred through purposive and snowball sampling, including young people between 18 and 29 living in urban and rural areas. Contacts with young migrants were established in person in public areas where migrant communities gather and live, such as certain parks and zones where migrants’ camps are settled. Contacts were also made by snowball sampling, by which young people recruited to participate gave further contacts of their peers. The final sample included 25 young migrants: nine Colombian, eight Venezuelan and eight Ecuadorian; 13 men and 12 women; 24 who identified as heterosexual and one as LGBTQI+. Regarding ages, 17 were 18 to 24, and eight were 25 to 29. Regarding migratory status, ten were undocumented and facing high social vulnerability at the time of the study, living in temporary camps or overcrowded housing in the cities of Iquique, Alto Hospicio and Pozo Almonte.

### Data collection and setting

Semi-structured individual interviews were carried out using an interview guide prepared by AO and AC. Each interview lasted between 45 minutes and 1 hour and was recorded for transcription after securing consent from the participant. AO, AC, and CD carried out the interviews. All had extensive previous experience carrying out research with international migrants.

All interviews with young migrants were conducted face-to-face in public places agreed between the parties. Interviews with health workers were conducted face-to-face in their workplace or through Zoom from their homes or workplaces. Data saturation was discussed among the team, and it was agreed to have been reached according to the research objectives of the overarching study.

### Data analysis

Interviews were audio recorded and transcribed verbatim onto a Microsoft Word document, and each transcript was checked for accuracy against the original recording. AO and AB carried out a separate inductive thematic analysis of the interviews, identifying patterns and themes derived from the data. Each researcher familiarised themselves with the data by first reading the transcribed interviews. Then, they carried out an open coding process by identifying concepts connected by similarities or differences within the raw data [41]. These concepts were then translated into codes and subcodes within overarching categories. It is important to note that the coding process does not respond to the quantitative logic of how much data a theme or code represents but rather its congruence with and contribution to the study’s primary objective [42]. The researchers then reviewed the main categories, codes, and subcodes each had identified separately. They undertook a consensus-building process, discussing discrepancies and agreeing on a final version.

The verbatim quotations selected for this article were translated from Spanish by a native English-speaker translator and checked by authors to verify the translations had captured their original meaning.

### Research team and reflexivity

A multidisciplinary team, including three social anthropologists, a political scientist, a psychologist, and a social epidemiologist, conducted the study. Each contributed to the study from their training and experience, and all have been conducting research with international migrants for several years. Conducting research as a multidisciplinary team allows for a critical approach to the issue studied, as different disciplines challenge each other’s established paradigms. The authors come from different ethnical backgrounds, and three of them have been international migrants themselves. Although this facilitates the team’s sensitivity on the topic of migration, it is crucial to recognise the privileges that accompany our position as women in academia, which could influence how the data was collected and analysed. To ensure that the study was being guided by cultural sensitivity and was appropriate to the contexts studied, we carried out regular meetings to discuss progress in detail and decided that CD would carry out the interviews with undocumented migrants, given her extensive work as a psychologist in such contexts in the North of Chile.

### Ethics

The overarching study followed the relevant guidelines and regulations for research involving human beings, including the Declaration of Helsinki. It was approved by the Ethics Committee of the Universidad del Desarrollo (#2019-22). Participation was voluntary, and participants filled out an informed consent form available online or on paper for participants with limited internet connectivity before taking part in the interview, securing written informed consent. All data were recorded anonymously, and no information allowing identification of the participants was kept except for the consent forms, which are held in the PI’s computer in a locked file.

## Results

The results are presented around four main themes: (i) legal, administrative, and organisational barriers, (ii) barriers derived from the healthcare system’s approach to SRH, (iii) stigma and discrimination, and (iv) facilitators for young migrants’ access to SRH. Table 1 shows the main results of the study.

**Table 1:** Barriers and facilitators that young migrants experience to access SRH services

Barriers (i) Legal, administrative, and organisational barriers • Health sector’s human resources: lack of available resources to ensure enough SRH specialists; high turnover rates of healthcare providers in general, and especially in rural and remote areas (experienced by the general and migrant populations) • Temporary ID sometimes asked as a requirement to access healthcare (illegal) • Lack of updated training for health workers in intercultural health and migrants’ health rights • Migrant’s fear of deportation limiting seeking healthcare (ii) Barriers derived from the healthcare system’s approach to SRH • Lack of comprehensive sex education in schools • SRH usually reduced to women’s reproductive health and pregnancy prevention • Health indicators required and evaluated without cultural specificity or regard to the characteristics of the population • Prevailing narrative locates the lack of prevention at the level of individual responsibility, neglecting structural factors, which is exacerbated in the case of young migrants • Little consideration for the role of men and their reproductive health needs • Heteronormative approach to SRH (iii) Stigma and discrimination • Perceived precocious initiation of sexual activity among foreign youth • Sexualisation of young migrants based on racial stereotypes • Foreign youth perceived to be engaging in risky sexual behaviour, which serves as a justification to explain unplanned pregnancy, STIs or HIV • Fear of testing for STIs and HIV among migrant youth • Obstetric violence exacerbated in young migrant women for being migrants	
Facilitators • Good healthcare experiences: receiving good treatment from healthcare providers, friendly and respectful care • Accessible and easily understandable information on SRH • Easy access to condoms • Social capital regarding sex education among young migrants • Community interventions and initiatives by health workers targeting migrant communities

(i) ***Legal, administrative and organisational barriers***

The results show several barriers that hinder access to SRH services for the general population, including migrants. Firstly, the lack of available resources to ensure enough SRH specialists was highlighted, in addition to the high turnover rates of healthcare providers in general, especially in rural and remote areas. This severely limits the range of services offered, including essential care. Limited human resources make it very difficult to provide comprehensive care, leading to long waits to access care, as reported by some of the young migrants interviewed. These problems were exacerbated during the COVID-19 pandemic, given that most resources were used in response to the health crisis, and face-to-face care was reduced to a minimum.

A barrier specific to the migrant population is related to the perception that a residence permit is a condition to access healthcare. Although this is not supported by the law, and healthcare access should be universal, several young migrants reported cases in which they were denied healthcare access for not having a temporary residence permit, as the following testimony by a young pregnant Ecuadorian woman illustrates:

The two previous times, we didn't know how to get to the hospital with my mum and my partner, it took us five hours walking all over Iquique. And many times, they didn't want to see me, because I didn't have a temporary ID and people at the health centre or wherever I was, told me that I had to have a temporary ID to get good care because, unfortunately that's how it was. (Woman, 25, Ecuador)

In another case, a young Colombian woman who arrived in the country 2 months pregnant was denied care for not having the provisional ID:

They told me they couldn’t help me if I didn’t have my papers. So, I had to go and file all the documents and everything and then I came back, but then about another month had passed, I was already 3 months pregnant. And the new person who assisted me told me, ‘you should have come earlier’, and I explained what had happened. Then he said that shouldn’t have happened… But, you know, you trust the person who is there, is qualified and should have the will to tell you things as they are. (Woman, 29, Colombian).

As we see in this last quote, in some cases, undocumented migrants’ access to healthcare depends on the will of the reception staff at health centres. According to the interviewees, this may happen due to healthcare providers’ lack of information about the rights of international migrants or because of outright discrimination. These experiences contribute to reinforcing the false idea that a residence permit is a requirement to access healthcare, which is common among the young people interviewed, as a midwife pointed out:

Of all the migrants, I think it is more about facilitating and doing more promotion to make it easier for them to access, because many of them don't know or have no idea that they have access, even if they don't have an ID. I think that, basically, they are going to have access as long as they know, because otherwise, they know that they are not welcome anywhere, why? Because they don't have an ID, because they are going to be asked for information, and they don't want to give themselves away, or for fear of being deported, they have many doubts about that. So, that is why they don't go either. (Midwife)

However, even when migrants are informed about their rights, care can be denied. This is known among migrants and fear of deportation represents an important barrier to access healthcare among those who have not regularised their migration status, as described by a psychologist:

They [undocumented migrants] have heard a lot of different experiences in the access to healthcare, and many prefer not to go to the health services, because they believe that just by accessing, they might be reported and expelled from the country. (Psychologist)

As we have seen, these barriers tend to be reproduced by health workers. This is the case because although regulations have become more inclusive, they have not been accompanied by training on intercultural health and migratory processes:

First of all, I feel that we, as health professionals, still have a long way to go in terms of accepting some of their cultural aspects, right? I think we have to raise awareness, train health teams. But also, the State, in general, needs training because there is a lack of understanding of what it means to be a migrant. No one is sensitised. Nobody. I believe that no one is currently sensitised to what migration is, the migratory process. (Midwife)

Raising awareness may contribute to reducing the difficulties in changing the idiosyncrasy of the healthcare sector concerning migrants, characterised by bureaucracy and excessive administrative procedures that hinder access to health for migrant communities in general and young people in particular. It may also reduce the negative stereotypes placed on migrant populations, which are especially visible regarding sexuality, as discussed later in this section.

(ii) ***Barriers derived from the healthcare system’s approach to SRH***

The results show gaps limiting comprehensive SRH in Chile for both local and migrant young populations, with particularities for the latter. Firstly, most health workers mentioned a lack of comprehensive sex education in schools. Initiatives to provide sex education to children and adolescents in schools have been pushed back and demonised based on a denial of sexuality and punitive approaches to sexual behaviour. Although this challenge must be addressed beyond the healthcare sector, it has severe consequences for the SRH of adolescents and young people. Additionally, a restrictive approach to SRH still prevails in the healthcare sector, prioritising women’s maternal role over other aspects of the sexual health of the entire population, regardless of their sex and gender status. SRH is then usually reduced to reproductive health and, more specifically, to pregnancy prevention, especially teenage pregnancy, as established by the Health Goals of the National Health Strategy of the Ministry of Health. Furthermore, the prevailing narrative around this topic locates the lack of prevention at the level of individual responsibility, understood as lack of self-care, which is exacerbated in the case of young migrants:

There are very few [young migrant women] who take care of themselves, and if they do, they have already had a baby before 19 years old and already have a one-year-old child, because they come, they arrive here and they don't take care of themselves, apart from the fact that it is also difficult for them because they are irregular in the country, because they pass through unauthorised crossing points. (Midwife)

This topic was especially highlighted among the midwives interviewed, given that national efforts in this area have been focused on increasing early adherence to pregnancy check-ups, and curbing the rate of unplanned pregnancies. In that sense, the increase of migrant population with less use of the healthcare system, may be seen as a threat:

In my CESFAM (primary healthcare centre), most of the population is foreign, we have the highest number of foreigners in Iquique, and yet they [health authorities] demand that we have a rate, a good indicator in pregnancy admissions under 14 weeks. But, some [migrant women] have not had anything done for 30, 38 weeks and we cannot do anything about it. What do we do with that? There is no way to solve it. (Midwife)

We take care of everything, we want them to take care of themselves, we want them to hopefully not continue procreating because otherwise, it's more problems for us too, so the more we take care of them, the better it is for us. (Midwife)

As these midwives explain, the health system assesses each centre with indicators and goals that are not culturally relevant nor specific to the characteristics of its patients. Thus, the unmet health goals of the migrant population -especially in the case of those who face the most vulnerability, such as undocumented migrants- become a “problem”. Therefore, many health workers interviewed stressed the healthcare system’s role in exerting the necessary control to compensate for migrants’ perceived lack of self-care.

For young migrants, access to contraception and healthcare during pregnancy were described as central topics, and obtaining information and care in these areas was crucial for them. The following quotes show this interest in accessing care, which can result in positive or negative experiences.

The midwife was a man. I told him I wanted to get an implant, but there were no implants, and he recommended an injection. He told me not to take pills because I might forget them and that the three-month injection was more effective. He explained everything well to me, I thought everything was fine. (Woman, 20, Venezuela)

I wanted to change my method [contraception method], and it was excellent, very fast, they explained me everything. (…) It seems to me that here they are always willing with all kinds of people, whether Chilean or not, and I really liked that. (Woman, 26, Colombia)

Above all, for my baby, I don't want anything to happen to my baby. I have a life in my belly and I don't want anything to happen to my baby. (…) That's why it hurt me so much when they didn't want to give me the provisional ID, I hit myself in the head like crazy… I'm trying so hard and it doesn't work out the way I expect it to. (Woman, 25, Ecuador)

These young women wanted to access SRH care; in the first two cases, they had good care experiences. In the last case, a pregnant woman who had been denied care for not having the provisional ID, describes how she banged her head against a wall, given her frustration when they denied her the documents. So, when viewed from the young migrants´ perspectives, many of the stories of “lack of self-care” become stories of structural vulnerability, facing additional challenges in accessing SRH care services and hampering their compliance with the expectations of the Chilean healthcare system.

It is also important to mention a tension in midwives’ perception of the reproductive control of young migrant women. Among the midwives interviewed, hand in hand with the negative perception of the high fertility rate of migrant women, a more nuanced perception arises derived from the fact that the migrant population maintain the fertility rate of the country:

At least 55% of our pregnant women are foreigners, so that means, doesn't it, that the fertility rate in the region or in the country, is increasing, or at least being maintained, right? (Midwife)

Together with the focus on women’s reproductive control, another limitation of healthcare system l for a comprehensive SRH approach is the little consideration given to the role of men and their own reproductive health needs, as well as a heteronormative approach of SRH. These are common to the general population but exacerbated among migrants. The healthcare workers interviewed pointed out that in most patient-provider interactions, there is an assumption of heterosexuality without opening a conversation about sex and gender. They also recognised their lack of skills to provide care to young LGBTIQA+ people in general, and migrants in particular. and A social worker declared, “From my own experience, I think that the professional training is generally heteronormative.” And a midwife expressed that:

Of course, a lot of the LGBT population is indeed arriving, there are quite a few young people, and I think there are certain professionals who are shocked to see the issue of homosexuality, especially in women. There is a lack of training in what is transsexuality or trans diversity. (Midwife)

(iii) ***Stigma and discrimination***

In the previous section, a midwife commented that health professionals “still have a long way to go in terms of accepting some of their cultural aspects”, and a young pregnant woman described how her access to healthcare depended on the provider. A social worker adds that:

Many times, access for migrants has more to do with the person [health worker] than with the system. There are those who make it very easy and those who give a thousand arguments and don’t allow them even to register. (Social worker)

These quotes are a reminder that perceptions of migration vary, and many people experience discrimination in health care simply for being migrants. In this section, we will delve into several dimensions of the discrimination and stigma experienced by young migrants concerning their SRH.

Firstly, some of the interviews show that “culture” is in many cases used to reproduce stigmatising discourses surrounding the perceived precocious initiation of sexual activity among foreign youth, especially those from Caribbean countries, as expressed by a midwife: “It is also a cultural issue, remember that in Caribbean countries, the beginning of sexual activity is early, teenage motherhood is normal” (Midwife). This goes hand in hand with discourses surrounding the sexualisation of young migrants, which were implicitly and explicitly identified throughout many of the interviews and contribute to the construction of young migrants as more sexualised individuals than the local population. Some health workers pointed out that migrants from certain countries, especially Caribbean countries, are more “uninhibited” or “more sexual” than Chileans, which was negatively perceived:

Now, looking at sexualisation, let's say, of the body, of people, for example, especially Colombian women, right? Colombian teenage girls are pretty voluminous, but at the same time, they wear very low-cut clothes, right? So, at first, it was shocking, even to me, I said, “Look at this girl, what she's exposing herself to, unnecessarily”, but it's their way of life over there. We, at least here, even though the summer is long, normally we Iquiqueños are not like that, but in the meantime, almost a decade has passed, so I think people have adapted, even Chilean women are dressing like Colombians [smiles], so to speak. (Midwife)

More Venezuelans have arrived here, and I can tell you that they carry it, they are more uninhibited when it comes to dancing, the girls have big cleavages... showing their tummies, with dances that have quite a strong sexual connotation… they are more sexual than our Chilean youth. (Midwife)

This “sexualisation” of Caribbean youth is recognised as a negative stereotype, as shown by a social worker and a young Venezuelan man:

I have heard young people who complain of being stigmatised by healthcare professionals, they have heard phrases like: “Colombian girls are seductive, with their tight pants”; this is, that they are super sexualised. (Social worker)

I think this idea is common, yes, not only in Chile. One always imagines that in the tropics, everything is liberation and everything is sex, orgies, and madness, and often this is not the case. (Man, 23, Venezuelan)

This was also reflected in some of the migrant participants’ experiences, where women described being treated differently and harassed by men for being perceived as promiscuous:

Most people who see me think I am Colombian or Venezuelan. Maybe because they have this idea in their mind that a Venezuelan or a Colombian woman, because of what they see on social networks or see elsewhere, that these people are very liberal. The intention to approach and propose something to you is there, but knowing that you, even if you answer kindly and more so when you answer kindly, if you give a hint of kindness, they are going to talk, and they talk to you until you don't stop them or just walk away, they are still going to be there. You try to be nice to the person, but they get confused most of the time and it's not right. Because you can just say hello and that's it, but they want to go beyond that and invite you to something. (Woman, 24, Ecuador)

Any attempt at sexual empowerment is interpreted as being “too liberal”, conducing to risky behaviour that should be kept under check. Additionally, it seems to serve as a justification for sexual harassment or to explain unplanned pregnancy, contracting an STI or HIV, and thus blaming young migrants for these situations. These perceptions also carry a moral assessment of sexuality, pointing to migrant youth as being especially responsible for risks with regards to their sexuality. This approach further hinders their sexual and reproductive rights.

Regarding sexual health, healthcare workers reported that the main reasons for seeking medical attention among young migrants were sexually transmitted infections (STIs) and HIV, as a midwife declared: “They ask for HIV testing, the main topics of concern for young migrants are diseases” (Midwife). This is connected to the fear that many young participants showed concerning STIs and HIV, expressed through their fear of getting tested in the past or as a barrier to getting tested in the future, which the health workers also observed: “I think that, first of all, the patients, I think that some of them are very afraid to get tested. Some know they have the risky behaviour, or they know they have it, but they are afraid to be told “you have it” (midwife). Added to this was the stigma associated with young migrants due to the prevailing idea among the local population that they are the ones who bring HIV to the country:

There was a lot of talk… when they talked about these high rates of HIV among youth, they blamed the migrant population, because we were a commune where there were… many, many migrants entered. A lot of migrants, and they were, and they were associated with that, I’ve heard it here. (Midwife)

I have thought about it [getting tested], yes, but no, because, of course, the relationships I've had are few but always protected. Maybe once I wanted to do it, but I kind of said 'no', I chickened out. (Man, 18, Colombia)

Among all groups of participants, some perceived that young people, in general (not only migrants), have a low-risk perception surrounding HIV, or in other words, that they are “not afraid enough” of it, hindering prevention:

I don't think they see the seriousness of the situation about AIDS because they don't live it. I think they should at least experience a mild infection for them to be afraid. Because they talk about AIDS, but we don't take care of ourselves when we are young. (Woman, 21, Ecuador)

STIs and HIV prevention thus hinges on emphasising risk and causing fear, bringing us to concepts of self-care and control similar to reproductive health and pregnancy. Self-care, again, is presented as an individual responsibility. In some participants’ words, a lack of self-care is conceptualised as promiscuity, which requires further control. This is especially concerning as some participants -health workers and young migrants alike- linked HIV, STIs and risk to LGBTIQA+ populations, especially men who have sex with men:

So, sometimes you don't know who you're dealing with and having sex with a man can be dangerous, because we can't see if he has a disease, if it's not HIV, it's something else. Lately we have seen a lot of gay men having this problem of sexually transmitted diseases, so I think teaching about sexuality is a very good thing. (Man, 29, Venezuela)

Considering that the migrant participants expressed fear as a barrier to seeking testing, these approaches are unlikely to successfully prevent STIs and HIV while conceptualising and emphasising sex as a risky activity and young migrants as at-risk individuals for themselves and others.

The production and reproduction of these stereotypes and stigmas about migrant youth among health workers translate into experiences of discrimination and mistreatment during healthcare, which is explicit in young migrants’ testimonies: “Even if we arrive the first, we are left for the last [to receive care]; that is called discrimination” (Man, 21, Venezuela), “There are many [health workers] that, when they see you are foreigner, they violate your integrity” (Woman, 29, Colombia). Young migrant women interviewed mentioned incidents of mistreatment in prenatal and childbirth care:

Now, with the pregnancy, they have told me that I have to be very strong because here in Chile, they say things to migrant women, especially when they arrive, crying a lot, complaining a lot. They tell us that we migrants complain too much and scold us over it. (Woman, 19, Ecuador)

For example, I was peeing, and at the time, I could not get out of bed because I had recently had a cesarean section, I had to wait. They left me waiting for three hours. Twice I told them, “I need to go to the bathroom”, but they looked at me as if I didn't exist and left me waiting for three hours (Woman, 23, Venezuela)

These quotes talk about obstetric violence being exerted on young migrant women. Although the local young population also reports these experiences of reproductive violence, they are exacerbated in migrant women since healthcare workers often single them out for being foreigners. Here, again, a moralistic approach to sexuality overlaps with xenophobia in a way that these abuses can be analysed as forms of punishment for transgressions with regards to “adequate” sexual behaviour in moral terms, which are “worse” in foreign women.

Being a migrant, as we have seen, is perceived by health workers as a risk factor regarding the SRH of young people. And although most testimonies stress the individual dimension of the risk, as the risky behaviours of young migrants, there are some who recognise migration as a structural adversity which puts young people at greater risk: “In general, migration is already a risk. They don’t come with the same resources, they don’t come with the capacity to guarantee their own wellbeing. But in general, the first few months are riskier, and then they stabilise. Here, we provide them with access to health care, the check-ups are regulated, adherence is generated, so some risks are reduced. They are referred to (someone who educates them), so this is also addressed. In general, I think the first few months come with some kind of risk.” (Social worker).

This last quote recognises the problem as one of structural vulnerability, which can be addressed through timely access to healthcare, as the cases presented in the next section will show.

(iv) ***Facilitators for young migrants’ access to SRH***

The experiences of discrimination mentioned above are reported mainly by young migrants who have recently entered the country and have an irregular migratory status. The young migrants interviewed who have been in the country for more years and have a regular migratory status usually offer a more nuanced account of their experience with the healthcare sector. This does not imply that they do not experience discrimination or mistreatment but that these are exacerbated among those who face higher degrees of migratory vulnerability.

Along with the above, it is critical to highlight that some young migrants and healthcare workers account for several aspects that facilitate SRH care for migrant youth. The first, and most named by young migrants, is good experiences in healthcare, which encourages them to seek care again when needed. These good experiences are described as receiving good treatment from healthcare providers, as well as friendly and respectful care where their privacy was safeguarded. The second most mentioned aspect was the provision of information by healthcare workers on SRH in a way that was accessible and easily understandable:

Yesterday, when I went to the doctor, the midwife explained things to me, she spoke to me well, that is, they know how to explain, they define things to you, they tell you what you have to do. I think so because I also saw in the hospital that there were a lot of posters about sexuality. (Woman, 21, Colombia)

With pregnancy, in the check-ups, it actually went very well, they took good care of me, when I had problems, they gave me a prescription and I never had issues or anything. (Woman, 24, Colombia)

These people were professionals, well, I was with professionals, they knew how to treat people. (Man, 29, Venezuela)

Several participants especially valued being able to easily access condoms, among other protection through the health system. A midwife interviewed reported how important it was for her that the health system facilitates access to protection for young migrants, both because it is a right and a good strategy for the protection of their SRH:

Another thing is consistent and effective access to condoms. If we do not guarantee this, then we simply do not know, one, how these sexual practices are being exercised, and two, how these people have or do not have access to effective information about this, which is something basic in prevention. Because, probably, the messages that we are, where we are directing the messages, are not the channels for these populations. For example, in national public television, these populations have very little access to television. (Midwife)

Another facilitator health workers identified was the social capital regarding sex education with which some young migrants arrive in Chile. On this topic, they highlighted, for example, how informed young Venezuelans were in terms of SRH compared to locals:

Colombians have more knowledge about sexuality. They talk to them from a very young age, I get the impression that they talk about sex at school, right? Because they know a lot and, not because of what they read on the internet, right? But because of what they have been taught. (Midwife)

However, this is not to be generalised, as there are also testimonies from migrant participants about not having openly discussed sexual health within schools of families, as a young Ecuadorian woman pointed out:

Yes, there are diseases, but young people will also not tell their parents or teachers that they have something, because they were not taught and the fear of being scolded for not knowing. They never sat down, “you know that's how you get it...”. Schools avoid that. It's a very big taboo, they avoid talking about anything related to sexual relations. They barely talk about women's menstruation because it's compulsory, but that's as far as knowledge goes. (Woman, 24, Ecuador)

Additionally, interventions carried out by health workers with migrant communities were reported as facilitators. These consist mainly of workshops in which information is given on various topics, including SRH. The highlight of these community initiatives is that, in addition to the delivery of information, they generate links between the health system and migrant communities, thereby breaking down some barriers that hinder young migrants’ access to SRH: “Let’s see, from the health team, at least when I worked in (healthcare centre), we used to do workshops, and the people who went most often were migrants” (Midwife).

While all these initiatives were identified as facilitators for SRH care as they contributed to bolstering young migrants’ agency over their SRH, they must go hand in hand with actions aiming at promoting migrant community-based and grassroots civil society organisations, which are the ones with the most access to hard-to-reach populations, as a young migrant activist explains:

And here, there is another phenomenon, talking about the general issue of HIV organisations here in Chile. First, they are, in my opinion, few. Secondly, the supply of services is also limited, compared to other neighbouring countries in the region. So, what this means is that, at least, the community-based organisations - which are the ones that have the most access to the population - are the ones that have the least access to resources, so it is the most paradoxical thing. The ones that have the most contact with the population are the ones that have the least access to resources, so, of course, in the overall picture, what this does is that, uh, we seem to be doing things backwards in terms of civil society and HIV. (Man, 26, Colombia)

## Discussion

This study explored barriers and facilitators to young Latin American migrants’ access to SRH care in the Tarapacá region of Chile, according to their perceptions and that of healthcare workers. In this last section, we discuss the study’s main results through four lines of analysis: the hegemony of reproductive health in the SRH of migrant youth, culture and othering, risk and structural vulnerability, and moving towards a comprehensive approach to SRH.

### The hegemony of reproductive health in the SRH of migrant youth

There is a particular focus on reproduction and birth control in some of the testimonies presented in this study, where reproductive health becomes little more than birth control for women. Furthermore, emphasis is put on women’s responsibility to avoid unplanned pregnancies. This can be analysed through the lens of Donzelot’s policing of families [43], which, in the context of reproductive health, implies responsibilities that are disproportionately imposed on mothers, according to the analysis of Calquín and Guerra. The same authors, furthermore, characterise the phenomenon as “neohygienism”, a term used to describe the neoliberal management of the health of vulnerable groups, or in the context of this article, international migrants [44]. Additionally, reproductive health is approached by the Chilean healthcare system from a heteronormative lens, leaving aside, on the one hand, the responsibilities and needs of men, as observed in another study [45], and on the other hand, lacking the skills and knowledge to address the SRH of LGBTIQA+ migrants adequately [46]. These issues have also been identified at the regional level in Latin America, hampering a comprehensive, impactful approach to the health and rights of migrants, regardless of their gender and sexual orientation and maintaining health inequities [19, 47].

### Culture and othering

While many challenges observed throughout the results presented in this study may be shared between locals and migrants, especially challenges linked to the Chilean healthcare system’s approach to SRH, young migrants face challenges of their own. In addition to barriers to accessing the healthcare system and SRH, which have been observed in other studies [48–50], culture kept emerging in the interviews with the healthcare workers who used it to explain some differences in negative terms. The participants, in their discourse, tended to adopt an “us versus them” narrative, where Chileans were described to behave in a specific (usually better) way and migrants in another (usually worse), constructing discourses of othering.

This was manifested by stigmatising discourses that were identified in some of the interviews with healthcare professionals, where migrants were sexualised. This was exacerbated with specific nationalities, for example, Venezuelan and especially Colombian youth who were stereotyped as “liberal” and “knowing too much about sex”. In contrast, Chilean youth are described as more conservative in this respect, even though there is also a moralistic view of sexuality towards them. This stigmatising and sexualised approach to migrant youth is present in society as a whole, promoting harassment and other violent behaviour towards, mainly, young migrant women. This issue is reported in a study by Pavez-Soto and Acuña [51], who investigated the experiences of street harassment and gender-based violence suffered by migrant girls and adolescents in the city of Antofagasta, Chile, concluding that today’s patriarchal society places migrant girls in a place of subordination, exposing them to situations of violence and harassment in public and private spaces.

Within health services, discrimination and stigma towards the migrant population can also contribute to exacerbating gender-based violence, as in the perpetration of obstetric violence. In the country, although this type of violence is experienced by women regardless of whether they are migrants, it is exacerbated and occurs in its most severe forms for women from vulnerable social backgrounds, including migrant women [52]. If we understand obstetric violence as a form of gendered disciplinary power that seeks to domesticate female bodies to behave in the ways that patriarchal society expects [53, 54], it makes sense that migrant women are especially targeted given their perceived exacerbated sexuality, as shown in the results section of this article.

In this context, promoting the SRH of migrant youth must include structural changes in narratives surrounding Latin American migrants in Chile, in political discourses, in the media, and in society in general. At the level of the healthcare system, intercultural training must be reinforced, including instances of examination and deconstruction of the stereotypes produced and reproduced. This would contribute to a more positive, intercultural approach to SRH, reducing health inequities in that area.

### Risk or structural vulnerability?

A third line of analysis of the results of this study revolves around risk and structural vulnerability. On the one hand, structural vulnerability is derived from structural violence, a concept first used in social sciences defined as “the indirect violence built into repressive social orders creating enormous differences between potential and actual human self-realisation” [55]. In the context of health, the broader term “structural vulnerability” includes the economic, material, and political insights of structural violence that encompass more explicitly cultural and distinctive sources of physical and psychodynamic distress [56]. On the other hand, connected to Lupton’s definition of risk as a pivotal discourse in normalisation strategies, discourses of control were identified in some of the participants’ testimonies, in which some individuals were labelled as “at-risk” individuals [36]. These individuals, because of they are perceived as inherently vulnerable as Latin American young migrants or because of their ungoverned behaviour, again, as Latin American young migrants, must be either educated into self-governance or subjected to the stigmatising consequences. Thus, access to, and especially the use of, SRH care is seen as an opportunity for governmentality [57].

Through the concept of self-care, which many participants brought up, young migrants are made responsible for their own SRH. This focus on individual responsibility, in a way, serves the purpose of filling the void between the struggles to identify and recognise structural violence and the need to find an explanation for SRH needs and outcomes in a population group seen as particularly at-risk but also as a threat to themselves and the public health of the “receiving” society. Rather than questioning and deconstructing harmful dynamics and discourses, the focus is on changing the individual behaviour of young migrants, appealing to self-control and self-governance, further deepening existing inequities. This lack of focus on structural forces driving risk and the emphasis on cultural characteristics and individual responsibility contributes to discourses that construct and reproduce stigma surrounding immigrant youth and SRH. In the context of migration, the concept of structural vulnerability is particularly relevant as it requires paying close attention to the effect that migratory regimes (laws, regulations, policies, and institutions) have on the SRH of migrant youth. However, it must go hand in hand with an action-oriented perspective aiming at promoting the agency and facilitating the empowerment of migrant communities regarding their SRH, along with profound structural changes. Otherwise, we may very well get stuck in a paralysis of sorts, where inequities are found to stem from such deeply engrained dynamics that no change or improvement is ever possible.

Along with the aspects just mentioned, it is worth highlighting positive actions identified by young migrants in relation to SRH, including friendly and respectful care and accessible and easily understandable provision of information of health care centres. These two elements can be interpreted as efforts made by the health system to promote their decision-making power and sense of agency over their SRH.

### Towards a comprehensive approach to sexual and reproductive health and rights

Finally, in the context of the study, it is necessary to discuss acting to promote the SRH and rights of young Latin American migrants to reduce health inequities in Chile and Latin America. For this, the framework put forward by the Lancet-Guttmacher Commission on Sexual and Reproductive Health becomes useful. It has been used before to analyse the SRH and rights challenges of South-South migrant girls and women in Central America and Mexico [58]. This framework includes a positive approach to sexuality and reproduction and establishes that achieving SRH depends on realising sexual and reproductive rights. From a human-rights perspective, these rights include having bodily integrity, privacy, and personal autonomy, freely defining one’s own sexuality and gender identity and expression, deciding whether and when to be sexually active, choosing one’s sexual partners, and having safe and pleasurable sexual experiences. Another critical component is guaranteeing access to the information, resources, services, and support necessary to achieve all the above, free from discrimination, coercion, exploitation, and violence, throughout the lifecycle [59]. Based on the study’s results and within the framework of the Lancet-Guttmacher Commission on Sexual and Reproductive Health, some action-oriented recommendations to advance the SRH and rights of young migrants can be made. Among them, questioning and deconstructing stigmatising discourses on young migrants and SRH at society, policy, and health system levels; designing and implementing inclusive, non-stigmatising intervention to promote all aspects of SRH in populations of all gender and sexual identities, specifically focusing on young migrants; providing good-quality and culturally relevant information through different, easily accessible platforms; promoting intercultural dialogue between young migrant patients and healthcare providers to foster safe spaces to address SRH needs; and efficiently and effectively training health professionals in SRH issues of migrant youth, integrating approaches to interculturality, gender and intersectionality. These gaps must be addressed to ensure that the reproductive and sexual rights of the whole population, regardless of nationality, are achieved and guaranteed. Along with the above, interventions must consider the working conditions of health workers in places with a high presence of migrant communities, where there is a high demand for care and the number of providers is sometimes limited. Additionally, it must be considered that healthcare workers do not always have access to current regulations regarding international migrants’ access to care. Finally, promoting collaborative and participative work between the health sector and migrant communities is essential to move towards a more just, equitable, and inclusive health system for migrant youth.

### Limitations

Although this study contributes to the existing literature on the SRH of young migrants in the context of South-South migration flows, it presents some limitations. Because of the nature of qualitative data, the results show important insights from a sample of Venezuelan, Colombian and Ecuadorian young people in the north of Chile. However, they cannot be generalised to the whole population of young migrants in the country. Only migrants from Spanish-speaking countries in Latin America were included, leaving out the perspective of non-Spanish speakers in Latin America and of migrants from the Caribbean, as well as other continents. This study is also limited as it did not include LGBTIQA+ populations and migrant sex workers. Considering their growing presence among the migrant population in Chile [60, 61], further research should seek to include these groups explicitly. Finally, migrants constitute a hard-to-reach population, which was exacerbated because more restrictive migration policies were implemented during the research period. Despite the particularity of this situation, it can shed important light on critical points of health services and ways to be better prepared for future health crises.

## Conclusions

This article explored barriers and facilitators to young migrants’ access to SRH care in the Tarapacá region of Chile from the perspective of young Latin American migrants and healthcare workers. Consistent with the existing literature at the national and international level, the results show a deficient approach to the SHR of this population group and that young migrants face similar challenges as the local population, with additional issues linked to migratory status, discrimination, othering, and stereotyping. Addressing the SHRH of young migrants and reducing inequities implies addressing these issues through profound systemic changes and working towards promoting the SRH and rights of young migrant individuals and communities.

## Data Availability

The anonymised transcriptions are available upon request from the corresponding author.
